# Application of foam assisted water-alternating-gas flooding and quantification of resistivity and water saturation by experiment and simulation to determine foam propagation in sandstone

**DOI:** 10.1016/j.heliyon.2024.e25435

**Published:** 2024-01-28

**Authors:** Javed Akbar Khan, Jong Kim, Sonny Irawan, Karina Aryanti Permatasar, Patrick G. Verdin, Baoping Cai, Nurudeen Yekeen

**Affiliations:** a College of Mechanical and Electronic Engineering, China University of Petroleum, Qingdao, Shandong 266580, China; b Department of Civil and Environmental Engineering, School of Engineering and Digital Sciences, Nazarbayev University, 010000, Astana Kazakhstan; c School of Mining & Geosciences, Nazarbayev University, Nur-Sultan City, Kazakhstan; d Petroleum Engineering Department, Universiti Teknologi PETRONAS, Malaysia; e Energy & Power, Cranfield University, Cranfield, MK43 0AL, UK; f Petroleum Engineering Department, Edith Cowan University, Australia

**Keywords:** Enhanced oil recovery, FAWAG, Foam flooding, Foam front, Resistivity, Water saturation

## Abstract

Foam flooding by Foam Assisted Water-Alternating-Gas (FAWAG) is an important enhanced oil recovery method that has proven successful in experimental and pilot studies. The present study is carried out to monitor the movement of the foam front once injected into the porous medium. This study aims to investigate applications of resistivity waves to monitor foam propagation in a sandstone formation. In the present lab-scale experiments and simulations, resistivity measurements were carried out to monitor the progression of foam in a sand pack, and the relationships between foam injection time and resistivity, as well as brine saturation, were studied. The brine saturation from foam simulation using CMG STAR is exported to COMSOL and calculated true formation resistivity. A diagram was produced summarizing the progression of foam through the sand pack in the function of time, which enabled us to establish how foam progressed through a porous medium. A surfactant and brine mixture was injected into the sand pack, followed by nitrogen gas to generate the foam in situ. As foam progressed through the sand pack, resistance measurements were taken in three zones of the sand pack. The resistance was then converted into resistivity and finally into brine saturation. As foam travels through the sand pack, it is predicted to displace the brine initially in place. This gradually increases each zone's resistivity (decreases the brine saturation) by displacing the brine. Also, an increase in the surfactant concentration results in higher resistivity. Finally, a comparison of three different surfactant concentrations was evaluated in terms of resistivity results, water saturation, and foam propagation monitoring to obtain the optimum surfactant concentration involved in foam flooding.

## 1 Introduction

Once the foam is injected into porous media, it becomes possible to effectively sweep a porous medium and recover amounts of hydrocarbon from high and low permeability regions. However, foam is considered very slow-moving and has low durability, and how far and fast it can progress needs to be clarified. There are few studies dealing with the progression of foam in a porous medium have been reported to date. Recently, joint analysis of velocity and resistivity have been used to determine morphologies of the hydrate [1]. In this work, the foam movement is monitored experimentally through resistivity measurements. When the foam displaces the native wetting fluid of the porous medium, the saturation level of that fluid changes. Such a change can be interpreted to identify the progression of the foam and its ability to propagate. As foam progresses, it is common for the flood front of the foam to disintegrate after some traveling distance. It is, therefore, also necessary to identify the point where the foam disintegrates and establish its behavior upon disintegration. Experiments and simulations carried out in this work are based on the Baronia field characteristics, with a main objective focused on determining the distance of foam progression. Moreover, the factors influencing foam progression in a porous medium have been identified, and a method of foam monitoring is proposed. Resistivity wave is considered to investigate and predict foam propagation in the porous medium by conducting laboratory-scale core flooding experiments. In this study, foam is injected as FAWAG in the sandstone formation.

## 2 Background

In hydrocarbon production, the initial reservoir pressure provides only a small fraction of the energy needed to recover the hydrocarbons. Therefore, fluids are injected into a reservoir to improve sweeping efficiency. Gas injection has an advantage over water, resulting in lower residual oil saturation. However, due to the high mobility of gas compared to water and oil, the injected gas tends to bypass the oil zone due to viscous fingering instability. Counteracting channelling through high permeability zones is also a major concern during gas injection. Recently, CO_2_ injection has become a favoured enhanced oil recovery technique. However, there are limitations linked to gas injection. Foam is an alternative solution; it entrains CO_2_ and steam to circumvent these challenges [2]. As such, it controls the mobility of the injected gas through areas of high permeability in heterogeneous formations. Foam is a large volume of gas dispersed in a continuous liquid phase. So, surfactants are used to stabilize the bubbles and prevent coalescence. The success of the foam injection process depends on the proper understanding of foam propagation. Some parameters affecting foam propagation and stability are flow rate, foam structure, surfactant concentration, capillary pressure, temperature, wettability, interfacial tension, ion exchange, and saturation [3]. Knowledge of foam propagation makes it possible to adjust some parameters controlling the process to predict the expected recovery.

Foam monitoring in porous media is difficult to observe. Foam monitoring is predicted based on differential pressure, foam resistivity, saturation, and foam flow rate. Foam propagation in porous media creates a pressure difference. Pressure difference becomes one of the indicators that foam is generated and propagated in porous media. As foam involves gas and surfactant injection to generate, the high-pressure difference caused by foam across core sections builds up [4,5]. The minimum pressure difference of foam depends on the gas and surfactant pressure difference, which is much lower for gas and surfactant used in foam generation [6]. The foam's differential pressure has significantly increased compared to oil, brine, and nitrogen gas. Several experiments have been conducted to monitor in-situ foam injection into sandstone using resistivity [7,8]. They have reported the increasing resistivity value indicates that CO_2_ propagates in some locations from the inflow side to the outflow side. The increasing resistivity has two meanings, such as the rapidly increasing resistivity and the replacement of formation water by CO_2_, related to the water saturation and the gradual increasing resistivity after CO_2_ passes through. Recently, numerous techniques have been adopted to explore the effectiveness of different injection mediums in the structural change of the formation. As the acoustic emission (AE) technique is adopted to monitor damage initiation and evolution in situ, optical scanning and X-ray computed tomography techniques are used for off-line damage identification and quantification [9]. The AE counts of saturated sandstones tend to be more than ten times smaller than those of dry sandstones. The AE count peaks of saturated sandstones are much smaller than those of dry sandstones [10]. Microfluidic models have gained much attention because they easily facilitate the imaging study of in-situ foam [11]. However, to estimate capillary pressure in a model with a uniform etching depth. A technique has been used to estimate foam's water saturation and capillary pressure. Unlike microfluidics with a uniform etching depth, these model fractures have a variation of aperture [12]. Dual-energy X-ray Computed Tomography (CT) imaging is a proven method of studying three-phase flow experiments in a porous media [13,14]. CT imaging techniques have been applied during foam flow to monitor the fracture growth in shale and fluid saturations within the sand column and gain insight into the relationship between foam morphology and non-aqueous phase liquid (NAPL) recovery mechanisms [15,16].

Foam with high quality (least water content) has shown high performance in the well stimulation [17,18]. The presence of high water content degrades the mechanical properties of rocks [19,20]. Increasing water content decreased the uniaxial compressive strength, elastic modulus, friction angle, and rock cohesion to different degrees [21,22]. A study uses the Nanoindentation technique to show the alterations in micromechanical characteristics of shale matrix minerals before and after ScCO_2_ water treatment under high temperature and high-pressure conditions [23]. Moreover, the dynamic mechanical properties of water-saturated coal decrease with progressively weakened latitude, as does increasing porosity [24]. A micro and mesoscale coal geometric model was initially reconstructed based on X-ray CT imaging and 3D reconstruction in fracture coal, it shows that axial strain plays a major role in peak velocity changes, while the interaction of axial strain with radial strain mainly influences the steady flow [25]. With regards to the water security, the spread of unwanted fluids in groundwater, such as poly-fluoroalkyl substances (PFAS), their high mobility makes their distribution in the environment ubiquitous due to leaching into underground water, runoff into rivers and oceans and deposits in soils [26]. Electrical capacitance volume tomography (ECVT) can therefore it is a powerful approach to detecting water saturation in porous materials [27]. The propagation of groundwater is different in different environments as it moves from one point to another because chemical weathering and ion exchange control groundwater chemistry, in which Na is released with the replacement of Ca at the beginning of groundwater movement. Ca and Mg gradually replace Na through a reversible ion exchange [28].

Foam is easy to disintegrate due to the presence of oil that will destabilize foam, depending on the pressure and temperature applied. The presence of oil and gravity forces mainly causes its disintegration, and when the bubbles coalesce, the thin film between the bubbles gets drained [29]. Foam has several properties that affect the foaming generation and foam propagation in the porous media, such as physical and chemical properties, electrical properties, and mechanical properties. Based on the foam properties, electrical properties are the most affected properties related to the resistivity measurement. Electrical properties are defined as the properties that affect the electrical charge contained in foam, which contributes to the foam behaviour towards electrical properties. The electrical property consists of the surface potential, defined as the ability of charge in two phases to attract the opposite charges and repel the same charges [30]. The attraction of the opposite charges is called counter ions and creates the attraction forces. The declination of the same charges is called co-ions and creates repulsive forces [31]. The attraction and repulsive forces are included in an Electrical Double Layer (EDL), which appears on the surface based on anion or cation. The working principle of resistivity wave is based on the electrical current that passes through a formation as it contains water with dissolved ions. Resistivity data is produced from the fluids in the pore spaces and by the interaction between the fluids and the rock surface [32]. A nuclear magnetic resonance has also been used to estimate the resistivity index of a sandstone reservoir [33]. The theory of the resistivity calculation is obtained using Ohm's Law [34]; this is expressed through Equations (1), (2)).

(1) $$r=\frac{V}{I}$$

(2) $$R=\frac{V}{I}∙\frac{A}{L}=\frac{r∙A}{L}$$

Where R is the resistivity (ohm.m, Ωm), V is the potential difference across the sample (volts, V), I is the current (amperes, A), r is the sample resistance (ohm, Ω), A is the sample cross-sectional area perpendicular to the current flow (m^2^) and L is the sample length (m).

Resistivity logs are used to find hydrocarbon zones and formation resistivity and are essential for water saturation calculation. Resistivity measurement is employed to calculate the formation resistivity (true resistivity, Rt) and the formation water resistivity (Rw), which leads to the water saturation calculation using Archie's equation. Resistivity is generated through the formation due to the electrically charged fluids stored in the pores. The electrical resistivity in the pore space contains current that is carried by ions and depends on porosity, pore fluid resistivity, salinity, fluid concentration, temperature, and pressure [35]. Moreover, micro-porosity significantly affects the electrical properties of porous media [36]. At the laboratory scale, resistivity measurement is performed using four electrodes mounted in the porous media.

Typically, one pair of electrodes has negative and positive charges, but four electrodes are used to avoid erroneous measurements, providing more accurate and frequent measurements. All four electrodes (E1, E2, E3, and E4) are connected to the data recording system. Each zone has two electrodes (positive and negative charges). Two current electrodes (C1 and C2) supply constant current in the porous media mounted in the bottom and upper parts of the core sample [2].

Resistivity measures the electrical properties of the porous media and the fluid stored in that formation. Resistivity occurs when the electrical field interacts with the charged surface immersed in the electrolyte solution. The resistivity measurement is also useful in evaluating [37,38]. The resistivity in porous media is defined as the movement of EDL from the charged surface to the liquid passing through the pores [39]. Resistivity measurement records the resistance of the electrical current flow in the rocks [40]. Rock matrix surfaces with charges and fluid movements stored in pore spaces commonly have different charges, causing pressure gradients and electrical current generation [41]. The electric current is generated inside the formation, which consists of cement, grain, and matrix. Electrical current flows through the interstitial pore structure of the formation. The working principle of electrical current in formation and various sedimentary rocks and resistivity profiles have been studied previously [42]. The resistivity of the formation is one important parameter for determining the hydrocarbon zone and type of formation. In reservoirs, sediment rocks and minerals are heterogeneous. Each type of sediment rock has a different wide range of resistivity profiles. The differences in resistivity profiles depend on the sediment rocks' material, the formation water's resistivity, and pore structure. It is thus possible to detect the formation and fluids inside the reservoir through resistivity logs.

This research focuses on monitoring the foam propagation using resistivity measurement in the porous media. Experiments use three different surfactant concentrations (0.1, 0.5, and 1 wt%), brine with 3 wt% of salinity (∼30,000 ppm), Baronia oil, and nitrogen gas. The characterization of fluids in static and dynamic conditions at ambient temperature (25 °C) and for a reservoir temperature of 90 °C are studied first; this covers viscosity, density, and resistivity. During the core flood experiment, foam propagation was recorded using resistivity measurement based on the fluid resistivity difference. The comparison of three different surfactant concentrations is evaluated in terms of resistivity results, water saturation, and foam propagation monitoring to obtain the optimum surfactant concentration involved in the foam flooding. The goal is firstly to present an advancement of the experimental procedure to measure resistivity and, secondly, to develop a theoretical framework for numerical simulations to be applied to predict the foam propagation behaviour. This study focused on developing the numerical model of responses during foam flooding using 3D reservoir simulations. The diffusivity equation is coupled to conventional reservoir simulators and then adjusted using the lab-based resistivity measurement values. In addition, interactions between foam injection time, foam propagation distance, foam velocity and foam flow rate using resistivity wave have been investigated, along with the effects of different foam concentrations towards resistivity.

The present study has been performed to investigate the possibility of applying the resistivity conversion phenomenon to monitor foam propagation during the foam flooding process. Seismic waves and electromagnetic waves have been applied to investigate the subsurface. Seismic waves have fair spatial resolution and good penetration depth. However, the impedance contrast between reservoir fluids is small. As such, they are insensitive to a reservoir's fluid content. On the other hand, electromagnetic waves have good electrical conductivity determination, but due to large wavelengths, they have poor spatial resolution. In this study, the electromagnetic wave is generated due to seismic wave excitation, a phenomenon called resistivity conversion, which is examined to monitor foam propagation. Such an arrangement should exploit the merit of using seismic and electromagnetic methods individually. Accordingly, the experimental study has provided insight into which fluid, flow, and wave parameters should be controlled to implement the proposed technique successfully.

## 3 Theoretical development

The concept of saturation is based on Archie's equation. It is used to determine the saturation and analyze the correlation between the resistivity of saturated rock samples and fluid resistivity [43]. Several parameters are involved in determining the saturation in the pore space, such as the physical properties of rock, fluid resistivity, and rock-fluid resistivity [44]. The electrical resistivity of the saturated rock is a function of porosity, the saturating fluid, the rock's resistivity, and the fluid's tortuosity, along with the electrical path. High salinity fluids correspond to low electrical resistivity. As explained in Archie's equation, it is assumed that the fluid conducts electricity through the pore spaces [45]. Moreover, another study found that Archie's model can fit the experimental data from foam. However, the saturation exponent for a foamed gas–water system is consistently higher than that of a pure gas–water system [46]. The correlation of resistivity in monitoring foam propagation in core samples and the change of saturation during a foam injection is explained in Archie's Equation (3).

(3) $$S_{w}=\sqrt[{n}]{\frac{a}{\emptyset^{m}}\;\frac{R_{w}}{R_{t}}}$$

Where $S_{w}$ is the water saturation, n the saturation exponent, a the tortuosity factor, Ø the porosity, *m* the cementation factor, $R_{t}$ the resistivity of partially saturated core with fluid (ohm.m, Ωm), and $R_{w}$ the formation water resistivity or rock, fully saturated with formation water (ohm.m, Ωm).

### 3.1 Foam monitoring using resistivity measurement

Foam monitoring is predicted based on differential pressure, foam resistivity, saturation, and foam flow rate. Foam propagation in the porous media creates a pressure difference, which indicates that foam is generated and propagates. As foam involves the gas and surfactant injection to be generated, the high-pressure difference is caused by the foam across the sections of the core specimen [47]. Note that the differential pressure of foam is significantly higher than that of oil, brine, and nitrogen gas. Besides using pressure difference to monitor foam propagation, resistivity is used to predict foam propagation. The changes in resistivity reading indicate that another fluid propagates, and water formation is replaced by another fluid related to the water saturation in the core sample [7]. The water saturation data are obtained from the resistivity measurement. Resistivity reading depends on the fluid concentration and electrical charges contained in the fluid [8]. Based on the resistivity and water saturation data, relationships can be established to identify the fluid distribution in porous media and predict the fluid velocity and flow rate. The foam velocity and flow rate are calculated to identify the foam behaviour in the porous media. The injection rate of the displacing fluid (foam) significantly affects the displacement in porous media and the overall recovery of the displaced phase (oil) [48]. Surfactant concentration is the parameter that mostly affects the foam flow rate and velocity [49]. Foam velocity and foam flow rate are obtained based on propagation distance over time, multiplying the specific area with the resultant velocity.

### 3.2 Foam injection methods and gravity segregation

Foams can be injected into a reservoir mainly in two different ways: firstly, through an injection of both gas and surfactant solutions together (co-injection process) and secondly, via a cycle alternating the injection of gas and surfactant (surfactant-alternating-gas SAG process). A co-injection injects pre-generated foams in the wellbore to enter the formation. At the same time, SAG causes a fluctuation in capillary pressure by repeating drainage and imbibition and naturally helps the creation of fine-textured foams during the process [50]. Chemical flooding by surfactant flooding alone or in combination with CO_2_/N_2_/polymer/alkali are established EOR methods such as injection of surfactant–polymer (SP), surfactant alternating gas (SAG) and alkali surfactant polymer (ASP) [[51], [52], [53], [54]]. Any gas and water injection into an oil reservoir will face gravity segregation. Gravity segregation is the tendency of fluids to separate into different layers because of gravity force. This concept is also shown to be valid with foams. An increase in pressure gradient during foam injection greatly increases the traveling distances before segregation, improving sweep efficiency.

### 3.3 Effect of subsurface heterogeneity

One of the most intriguing foam properties in porous media is its capability to overcome subsurface heterogeneity. Permeability contrast, often regarded as the most important aspect of subsurface heterogeneity, leads to poor sweep efficiency resulting from early breakthroughs. Conceptually, the foam's capability to overcome the heterogeneity is endowed by its sensitivity to capillary pressure [55]. The low capillary pressure environment makes Foam films more stable in high-permeability layers. In contrast, the high capillary pressure environment makes foam films less stable in low-permeability layers. Foam films cannot sustain due to too high capillary pressure (foam films cannot survive due to lack of aqueous phase).

### 3.4 Gas mobility reduction

The foam flooding mechanism is mainly due to reduced gas permeability and increased foam viscosity. Commercial simulators, like COMSOL, model the foam behaviour using a mobility reduction factor (MRF). The mobility-reduction factor is defined through Equations (4), (5).

(4) $$k_{rg}^{f}=k_{rg}^{nf}\left(s_{w}\right)\times FM$$

(5) $$FM=\frac{1}{1+MRF∙F_{1}∙F_{2}∙F_{3}∙F_{4}∙F_{5}∙F_{6}∙∙∙}$$

Where, $k_{rg}^{f}$ is the relative gas permeability in the presence of foam, FM is dimensionless interpolation Factor, FM = 1 (no foam), FM = 0 (strong foam), F1 is the surfactant concentration term, F2 is the water saturation term, F3 is the oil saturation term (foam killer), F4 is the gas velocity term, F5 is the capillary number, F6 is the critical capillary number.

## 4 Methodology

In this research, foam propagation by resistivity measurement incorporates experimental lab work, simulation, and data analysis. Three main factors were considered: fluids, core sample, and electricity applied to supply the power system into the Inductance, Conductance, and Resistance (LCR) meter during the injection process.

### 4.1 Materials

The Idoha Cray sandstone was used as the core sample. This homogeneous core sample with 30 % porosity and 700–1000 mD of brine permeability was ideal for studying foam propagation. The sample was 0.3 m long and a diameter of 0.0381 m. The foam propagation success depends on the core sample specification and the equipment used. The equipment with the least percentage error would determine the most accurate result in the core flood experiment. A displacement test rig as core flood equipment is needed to monitor fluid injection in the core sample. This equipment is built by adding and improving some parts, such as the length of the core sleeve and the resistivity measurement, noting that most other core flood equipment reported in the literature do not use the resistivity measurement as a method to monitor foam propagation in porous media. Table 1 summarises the chemical composition of the Idoha Cray sandstone. The chemical composition shows that the Idoha Cray Sandstone mostly contains silica (SiO_2_), which has large negative charges, thus contributing to the resistivity measurement during the core flooding.

**Table 1:** Composition of Idoha Cray sandstone core.

Mineral composition	Values
SiO_2_	86.47 %
Al_2_O_3_	7.31 %
FeO/Fe_2_O_3_	1.14 %
TiO_2_	0.70 %
CaO	1.21 %
MgO	1.65 %
H_2_O	1.20 %

### 4.2 Chemical fluids

This section describes several fluids that generate foam in situ in the core sample. The foam was made through a mixture of fluids with different properties, all affecting the foam characteristics. The fluids, including 2 L of Baronia oil, Nitrogen gas, and Brine with 3 wt% salinity, were dissolved in 2 L of water and stirred using a magnetic stirrer. MFOMAX with 0.1 wt%, 0.5 wt%, 1 wt% concentration were prepared and dissolved in 2 L for each concentration. Several mixture solutions were prepared with different surfactant concentrations and fixed brine concentrations at 3 wt%. All concentrations were dissolved into distilled water and stirred using a low-speed magnetic stirrer.

### 4.3 Baronia/Bokor field petro physics properties

**Fig. 1:**
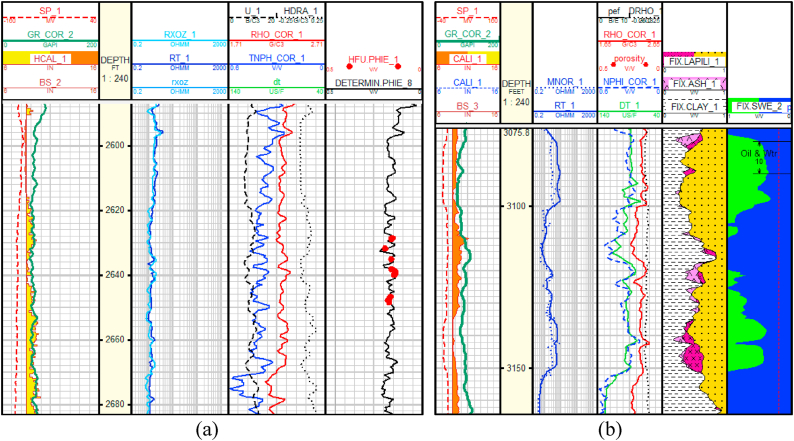
Log data of Well in the Baronia field, (a) Neutron Porosity Log (b) Water saturation estimation.

Table 2 shows the reservoir data in Baronia Field. The Baronia field (a BDO Field) is located 40 km offshore of Sarawak in the Southwestern part of the Baram Delta Province, Malaysia. The area is estimated to have more than 4000+ MMstb oil in place with multiple stacked sandstone reservoirs in a shallow offshore environment. They have been in production for more than 30 years. The historical production data indicated that the oil production has been relatively flat at 80–100 kbd of oil, primarily from infill drilling and new in-field development and rejuvenation. Currently, many wells are shut in due to the high water production. Because of the offshore environment, well spacing is relatively large, and the platform has limited available space. These characteristics present a challenging environment to carry out EOR operations. The latitude and longitude of the Baronia field considered in this work is 4° 45′ 00″ N and 113° 45′ 00″ E. The water depth is 250 ft below the Mean Sea Level (MSL). It is an upfaulted domal structure without internal faulting located between two East-West trending growth faults. The prospective sequence consists of sandstone interbedded with siltstone and shale of Late Miocene age at depths of 1615–2410 m (5300–7900 ft). The sandstone thickness ranges from 3 to 75 m (10–245 ft), with shale thickness within 105-90 m (5–295 ft) [56]. Fig. 1 (a, b) shows log data for porosity, water saturation and the results of gamma-ray logs are used to determine the distribution of sand in the reservoir. The green and yellow sand sections in gamma-ray logs define the poor and better-developed sandstones. The range of porosity within 15–26 % is located in the green section, and the 26–30 % range is in the yellow section. The sandstones are mostly fine-grained, and the permeability ranges between 100 and 350 mD [57]. Hydrocarbons in Baronia are found in stacked reservoirs. The salinity range is within 20,000–35,000 ppm. The main reserves are distributed over 10 sandstone reservoirs with at least 8 separate oil-water contacts. The Baronia crude oil is classified as light crude oil, which has 38.5–41° API, waxy (3–4 %), and is a land plant-delivered oil with low sulfur content (0.07 wt %) [57].

**Table 2:** Basic Baronia field reservoir data.

Parameter	Value	Parameter	Value
Boi	1.78	Datum Depth, fts MSL	7830
API Gravity	41.8	Initial Reservoir pressure, psi	3420
Average Permeability, md	70	Bubble Point pressure, psi	3380
Porosity, %	15–26	Reservoir Temperature, F	204

Porosity is determined using Neutron-Density log combination. There is a deflection between neutron and porosity log even though the separation is not far. Thus, correction is carried out to determine the porosity value. The formation water resistivity is calculated from the formation resistivity log, and Pickett-plot and water saturation are determined using Archie's resistivity relationship as shown in Equation (6).

(6) $$R_{t}=a\varphi^{-m}S_{w}^{-n}R_{w}$$

Where *m* and *n* are the cementation and saturation exponent respectively, and $R_{w}$ is the brine saturated rock resistivity. The values *a = 1*, *m = 2*, and *n = 2* used for this field are obtained from the laboratory measurement. The formation water resistivity is determined from the formation water salinity and the depositional environment.

### 4.4 Experimental procedure

This research incorporates experimental lab work, measurement, and data analysis. Important factors affect foam propagation in porous media, such as the core sample (Idoha Cray Sandstone), the chemical fluids used, and the electricity applied during the injection process. As mentioned previously in Section 4.2, experiments involve several fluids of 3 wt% of brine as the synthetic seawater, MFOMAX as the surfactant with three different concentrations (0.1 wt%, 0.5 wt%, 1 wt%), Baronia oil and Nitrogen gas. Before conducting foam injection into the core sample, fluid characterizations in static and dynamic conditions were conducted to analyze the fluid properties experimentally. Foam injection was performed using the alternating injection method to generate foam in situ. During this foam injection in the core sample, injection time and resistance were recorded using the LCR meter. When the resistivity measurement was complete, several parameters were calculated, including resistivity, water saturation, foam propagation distance, foam velocity, and flow rate. Based on the data input considered, i.e., the core sample properties, properties of the fluids used, injection parameter, and electrical supply for the LCR meter, the core flooding was generated, and output was collected and used to analyze the foam propagation time and distance, foam velocity and flow rate, and the water saturation.

#### 4.4.1 Resistivity measurement under static and dynamic conditions

**Fig. 2:**
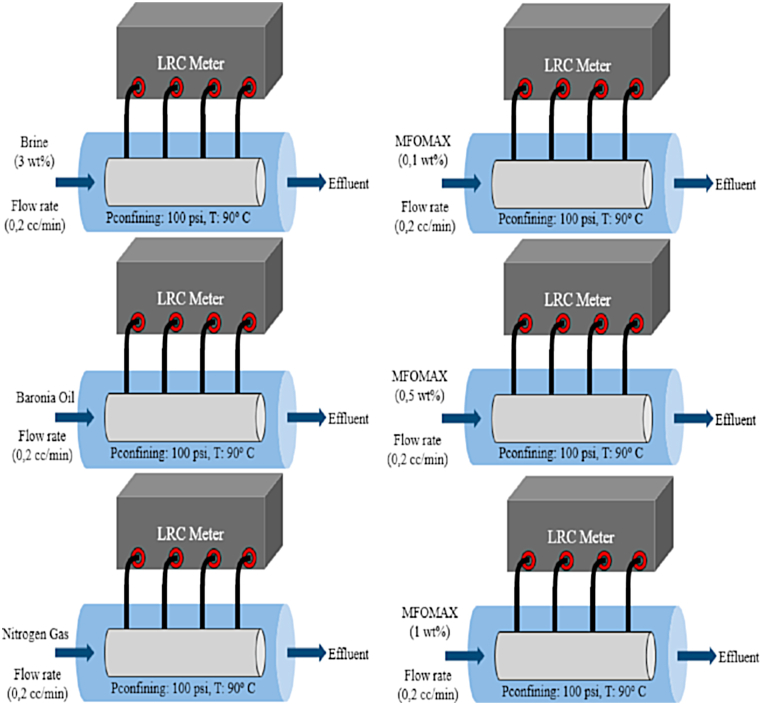
Scenario of dynamic condition experiment.

The resistivity measurement was used to monitor foam propagation in the core sample. Since the foam propagation is difficult to predict under such conditions because of the mixture of several fluids and core-fluid contacts, a fluid characteristic assessment was conducted under dynamic conditions. Before injecting the foam, each fluid was injected separately into the core sample to correlate its resistivity profile with the resistivity profile of the injected foam in function of time. The resistivity reading was used to establish the water saturation of the core sample and identify the fluid displacement due to the foam propagation. Static resistivity measurements were performed using a conductivity meter manufactured by Metler Toledo. 250 ml of each solution was placed in the beaker, and the electrode was immersed into the solution to measure conductivity and resistivity data simultaneously. Measurements were performed three times, and average values were taken. The injection process determined the dynamic condition via a displacement test rig operated for a temperature of 90 °C, an injection flow rate of 0.2 cc/min, and a confining pressure of 100 psi. These values were used as reference. Before the injection, 2 L of each solution were prepared. The core sample was placed into a rubber sleeve connected to the electrodes and the LCR meter. The core sample was then placed into the confining chamber and was heated to 90 °C using an oven attached to the displacement test rig at temperature. When the temperature reached stable, a confining pressure of 100 psi was applied to the core. Each solution was injected into the core sample at a flow rate of 0.2 cc/min until the differential pressure reached a stable state. All fluids were injected with the same procedure, and constant parameters were applied. Resistivity measurement was connected to the displacement test rig and recorded during the injection. Details of the experiments are presented in Fig. 2.

#### 4.4.2 Core flood experiment

**Fig. 3:**
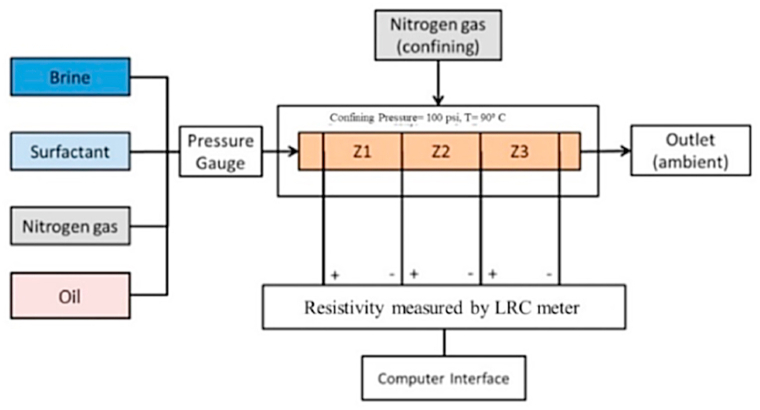
Illustration of experimental set up with different zones and resistivity tool.

The experiments and the in-situ foam generated by alternating Nitrogen gas and surfactant were carried out at 90 °C and an injection pressure of 50 psi. A schematic illustration of the experimental setup is shown in Fig. 3. All experiments with foam injection into the core sample were performed in the horizontal direction.

**Fig. 4:**
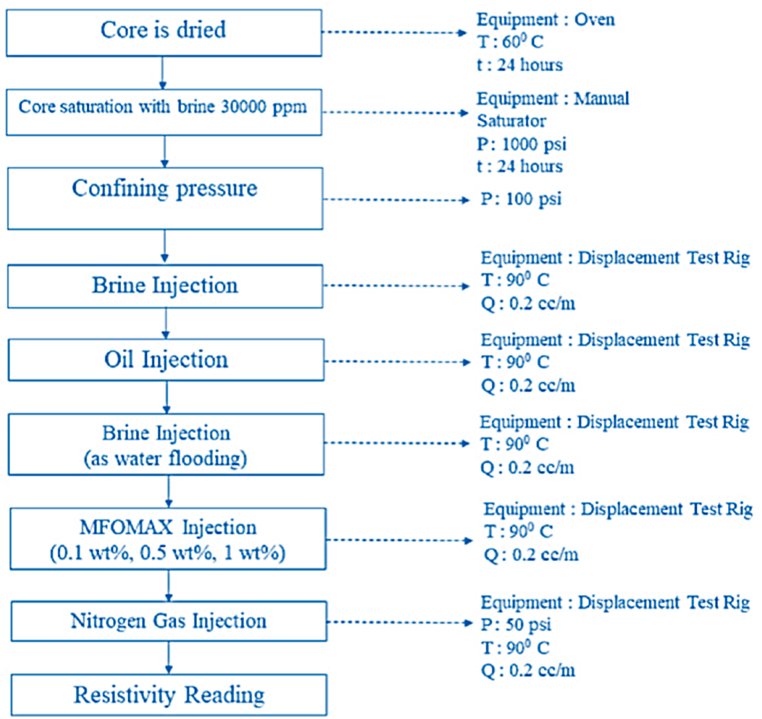
Core injection sequence.

The core flood experiment was conducted using several injection parameters, as specified in Table 3. The core flood experimental procedure details are presented in Fig. 4. The same injection parameters and the core flood procedure were applied to the foam injection.

**Table 3:** Injection parameters.

Parameter	Values
Brine Injection Rate (cc/min)	0.2, 0.5, 1
Injection Rate (cc/min)	0,2
Injection Volume	3 PV
Temperature (^o^C)	90
Confining Pressure (psi)	100
Nitrogen Gas Injection Pressure (psi)	50

**Fig. 5:**
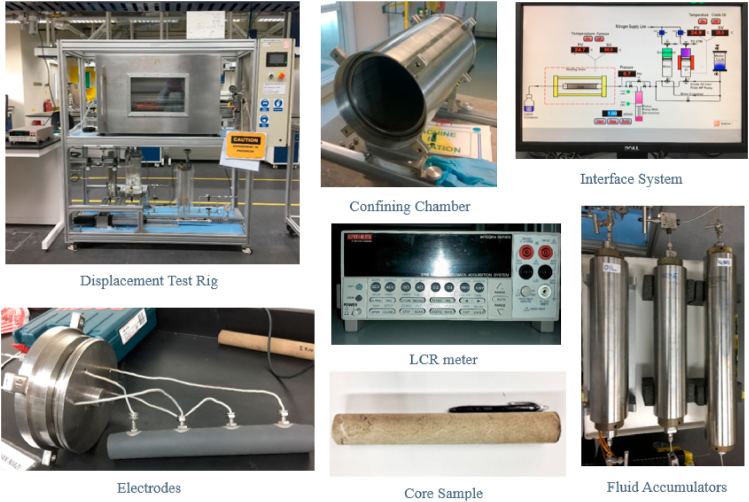
Core flood rig, material and equipment preparation.

The core sample was selected with high porosity to ease the foam propagation. The displacement test rig, as shown in Fig. 5, used for these experiments, consists of a confining chamber, a 0.3 m long core holder of diameter 0.03 m, four accumulators operating at 90 °C maximum temperature, a maximum pressure of 150 psi, a furnace with a maximum temperature of 110 °C, a pressure indicator, two and three-way valves, an LCR meter, an interface system, and four electrodes of copper strip coated with silver. All the core flood systems, software, and resistivity equipment were connected and formed the integration system.

The displacement test rig had several parts that needed to be connected. The first step of the displacement test rig installation was to load each solution into the fluid accumulator. Tubing was placed from the fluid accumulator to the inlet of the injection and the pump. All four electrodes from the core sleeve were connected to the LCR meter. This core sleeve was placed into the confining chamber. Porosity was obtained by measuring the length and diameter of the core sample using the digital Vernier caliper, and the core sample's bulk volume was calculated using the selected core's dimension. The core sample was initially dried using an oven for a minimum of 24 h, and the weight of the dry core sample was measured. The core sample was saturated by brine using a brine desiccator and under vacuum for about 24 h. The weight of the wet core sample was measured, and the pore volume of the core sample was calculated based on the weight difference using Equation (7).

(7) $$V_{pore}=\frac{Dry\;core\;weight-Wet\;core\;weight}{\rho_{fluid}}$$

The core sample was placed in the core holder. Two gas injection pressures were adjusted, the confining pressure at 220 psi and the upstream pressure at 400 psi. Porosity and permeability were then calculated automatically using the Poroperm software. Brine 3 wt% was injected into the core sample and circulated throughout the core sample at three different flow rates: 0.2 cc/min, 0.5 cc/min, and 1 cc/min to obtain the brine permeability. The differential pressure was monitored during the brine injection. The brine permeability calculation was determined according to Darcy's law in Equation (8).

(8) $$K=\frac{Q\mu L}{A\Delta P}$$

Where, K is the permeability, *Q* is flow rate of the injected brine, *μ* is the brine viscosity, *L* the length of the core sample, *A* the cross-sectional area of the sample, and ΔP the differential pressure between inlet and outlet of the core.

The LCR meter used in this experiment measures the electrical resistance in the core sample. The resistivity reading was carried out using a Keithley 2700 Integra Series LCR meter (Inductance Capacitance Resistance). This equipment measured resistance from four electrodes connected in the core sleeve. After resistance reading, the resistivity value was determined according to Ohm's law. Based on the datasheet, the general specifications for this equipment are a power supply of 220 V, power consumption of 28 VA, and an expansion slot 2. Foam injection was performed on the alternating injection to generate foam in situ. During foam injection in the core sample, foam injection time and resistance were recorded using the LCR meter. After the resistivity measurement data was obtained as time progressed, several parameters were calculated, such as resistivity, water saturation, foam propagation distance, foam velocity, and flow rate. Data input consisted of core sample properties, properties of fluids used, injection parameters, and electrical supply for the LCR meter. Based on the data input defined, core flooding was conducted, and data output was produced and used to analyze foam propagation time, foam propagation distance, foam velocity, foam flow rate, and water saturation. The summary of data input and data output are explained in Table 4.

**Table 4:** Summary of data input and output.

Data Input		Data Output
• Measured Core Sample Properties a. Dry Weight (g) b. Wet Weight (g) c. Cross Sectional Area (m^2^) d. Length (m) e. Core Plug Bulk Volume (cc) f. Cementation Exponent g. Saturation Exponent • Calculated Core Sample Properties a. Pore Volume (g) b. Porosity (%) c. Flow Rate (cc/min) d. Differential Pressure (psi) e. Permeability (mD) • Measured Properties of Brine a. Brine Salinity (PPM) b. Brine Density (g/cc) c. Brine Viscosity (cp) d. Brine Resistivity (ohm.meter) • Measured Properties of Surfactant a. Surfactant Concentration (%) b. Surfactant Resistivity (ohm.meter)	• Measured Properties of Baronia Oil a. API b. Oil Density (g/cc) c. Oil Viscosity (cp) d. Volume of Oil (cc) e. Oil Resistivity (ohm.meter) • Nitrogen Gas Injection Parameter a. Differential Pressure (psi) b. Injection Rate (cc/min) c. Nitrogen Gas Viscosity (cp) • Surfactant Injection Parameters a. Surfactat Volume to be injected (cc) b. Injection Rate (cc/min) c. Injection Time (min) • Brine Resistivity (Rw) • Constant applied current, I. • Distance of electrodes in the core sample (m) • Time to monitor foam propagation (min)	• Potential difference (V). • Resistance (r). • Fully saturated core plug resistivity (Ro). • Partially saturated core plug resistivity (Rt). • Brine saturation after injection (Sw). • Oil saturation after injection (So). • Resistivity (Rt) vs injection time (t) graph. • Brine saturation (Sw) vs injection time (t) graph. • Oil saturation (So) vs injection time (t) graph. • Resistivity (Rt) vs distance graph. • Diagram of brine saturation values at various time steps (monitoring) • Diagram of oil saturation values at various time steps (monitoring) • Foam propagation velocity and flow rate

### 4.5 Simulation procedure

In order to create a true resistivity domain for laterolog modeling, the water saturation from foam simulation using CMG STAR is exported to COMSOL. In this forward simulation, we calculate the true formation resistivity using Archie's equation. A finite element numerical modeling software package, COMSOL Multiphysics 5.3, was used to model laterolog response for foam flooding. The task was framed as a DC problem due to the low frequency of device measuring conditions. The two-dimensional asymmetrical model domain was discretized by triangular mesh. Laterolog device and its surroundings were discretized using the built-in automatic mesh function in COMSOL. So, this paper presents a finite element numerical model of a laterolog borehole probe used in practice. First, we compute the electrode coefficient of this tool inhomogeneous medium by using finite element method (FEM). Second, we conduct an extensive numerical investigation to determine the tool response to the foam front in a heterogeneous reservoir. Thus, CMG STARS software was used as a compositional reservoir simulator for foam flow simulation. The package allowed for defining the foam component, foam generation, and foam collapse rate. The simulation solves the complex numerical fluid and foam flow simultaneously. This study used an empirical approach to simulate foam flooding in the reservoir. In this approach, foam mobility reduction was mainly based on surfactant concentration. The procedure is explained below.

1. The simulation study was started by building a 3D grid to simulate foam flooding in a water-flooded reservoir.
2. The model simulated multiphase fluid flow through a heterogenous reservoir from injector to producer well. There were three methods of foam injection: pre-foam, co-injection, and surfactant alternating gas. In this study, pre-generated foam injection was used.
3. Then, a finite element numerical modeling software package, COMSOL Multiphysics 5.3, was used to model laterolog response for foam flooding.
4. The task was framed as a DC problem due to the low frequency of device measuring conditions.
5. The two-dimensional asymmetrical model domain was discretized by triangular mesh.
6. Laterolog device and its surroundings were discretized using the built-in automatic mesh function in COMSOL.

#### 4.5.1 3D model

**Fig. 6:**
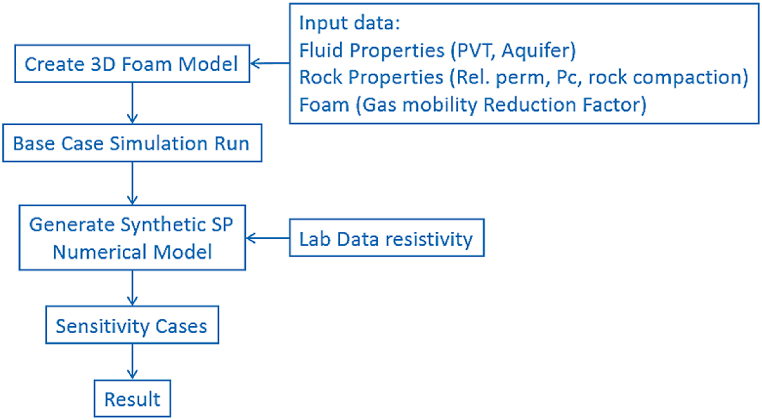
Process flow for streaming potential numerical model.

The two-phase flow was studied using a 3D model built in the COMSOL reservoir simulator; the process flow is shown in Fig. 6.

Field fluid properties and PVT calculation results were used as input data to the numerical model. Several models with varied electrical and geometric parameters were prepared to estimate the streaming potential (SP) responses in several cases. The data inputs are listed in Table 5.

**Table 5:** Data input for simulation.

Parameter	Value
Porosity	0.15–0.25
Permeability (mD)	60–100
Depth (ft)	7800
Reservoir Temperature (F)	204
Oil API	41.8
Reservoir Pressure (psi)	3400
Bubble Point Pressure (psi)	3380
Foam Gas	N_2_ + Surfactant

#### 4.5.2 SP numerical model

Resistivity data of the fluid from the laboratory experiment measurement were used to adjust the coupling coefficient to compute SP. Vertical SP profiles along the production well and lateral SP profiles were generated.

#### 4.5.3 True formation resistivity

To create the true resistivity domain for Laterolog (log to measure resistivity) modeling, the water saturation from the foam simulation was exported to COMSOL. The true formation resistivity was established using Archie's Equation (3). The parameters used in the Archie equation are listed in Table 6.

**Table 6:** Archie resistivity parameters.

Parameter	Value
a	0.6
m	2
n	2
Rw	0.15–0.25
Porosity	0.08

#### 4.5.4 Laterolog response finite element Modelling and finite volume method

COMSOL Multiphysics 5.3 was used to model the Laterolog response for foam flooding. The task was framed as a DC problem due to the low frequency of the device measuring conditions. A 2D asymmetrical model domain was discretized using a triangular mesh. The Laterolog device and its surroundings were meshes using the COMSOL automatic mesh function. The Laplace Equation was solved in the two-dimensional, asymmetrical, inhomogeneous model domain to simulate the operation of the Laterolog tool in DC mode. The steady-state equation is written in Equation (9).

(9) $$\frac{\dot{\partial }}{\partial z}\left(\frac{1}{R\left(r,z\right)}\frac{\partial V\left(r,z\right)}{\partial z}\right)+\frac{1}{r}\frac{\partial }{\partial r}\;\left(\frac{r}{R\left(r,z\right)}\frac{\partial V\left(r,z\right)}{\partial r}\right)=Q$$

Where *R*, *V*, and *Q* denote the resistivity, electric potential, and volumetric current density in the homogenous porous medium respectively; *r* and *z* are the radial and axial coordinates, respectively.

In order to calculate the apparent resistivity, i.e., the tool response given to the real geological situation, the probe coefficient must be determined. The probe coefficient for an inhomogeneous borehole tool with finite length can be determined when the probe is situated in a homogeneous medium (rock) with known resistivity (*Rt*), and the potential *Vm* is calculated numerically using Equation (10).

(10) $$K=R_{t}\frac{l_{m}}{V_{m}}$$

Owen and Greer estimated the three-electrode tool coefficient where the potential field has a prolate spheroid shape [23], as per Equation (11).

(11) $$K=\frac{4\pi b}{\ln\frac{c+k}{a}}$$

Where *a* and *b* are the radius and length of the electrode centre, and *c* is the length of the guard electrode.

The apparent resistivity $R_{a}$ of the three-electrode Laterolog is obtained by measuring the electric potential of the middle electrode, *Vm*, and the current discharged from the middle electrode, Im using Equation (12)

(12) $$R_{a}=K\frac{V_{m}}{l_{m}}$$

The finite element method was adopted to simulate the foam flow, and the CMG STARS software was used as the compositional reservoir simulator. The package allows defining the foam component, foam generation, and foam collapse rate. In this approach, the foam mobility reduction was mainly based on the surfactant concentration.

## 5 Results and discussion

The results consist of an investigation of foam resistivity as a function of time in various surfactant concentrations, water saturations as a function of time, and various surfactant concentrations, followed by the determination of the surfactant concentration, foam propagation monitoring, and recovery factor. An investigation of foam propagation monitoring encompassing resistivity measurement as a function of distance in various surfactant concentrations, foam velocity and foam flow rate.

### 5.1 Fluid and core characteristics

Table 7 shows that the Baronia oil has by far the highest resistivity compared to other fluids, indicating that only a few numbers of charges are present in it. The lowest resistivity value was obtained for the mixture of MFOMAX, brine, and Baronia oil due to the dominancy of the MFOMAX and brine molecules compared to those from the Baronia oil in the solution. Resistivity measurements of mixtures have been carried out three times to confirm the recorded data. Brine is known to be a conductive fluid with a higher number of positive and negative charges, indicating that brine can transmit the electrical current without other fluids involved. MFOMAX has a lower resistivity, close to the resistivity value of brine. The increasing MFOMAX concentrations cause lower resistivity. The higher the MFOMAX concentrations, the lower the resistivity because of the highest number of positive and negative charges in the solutions.

**Table 7:** Fluid properties at static condition and core characteristics.

Solution	Resistivity	Idoha Cray properties	
	(ohm.m)	Properties	Value
Baronia Oil (API:44)	28.9	Porosity (%)	30
Brine	0.0002552	Pore Volume (cc)	102.55
MFOMAX	0.0005908	Bulk Volume (cc)	341.85
MFOMAX + Distilled Water	0.06366, 0.01598, 0.008492	Brine Permeability (mD)	700–1000
MFOMAX + Brine	0.0002596, 0.0002562, 0.0002514		
MFOMAX + Brine + Oil	0.0002534, 0.0002504, 0.0002464		

**Fig. 7:**
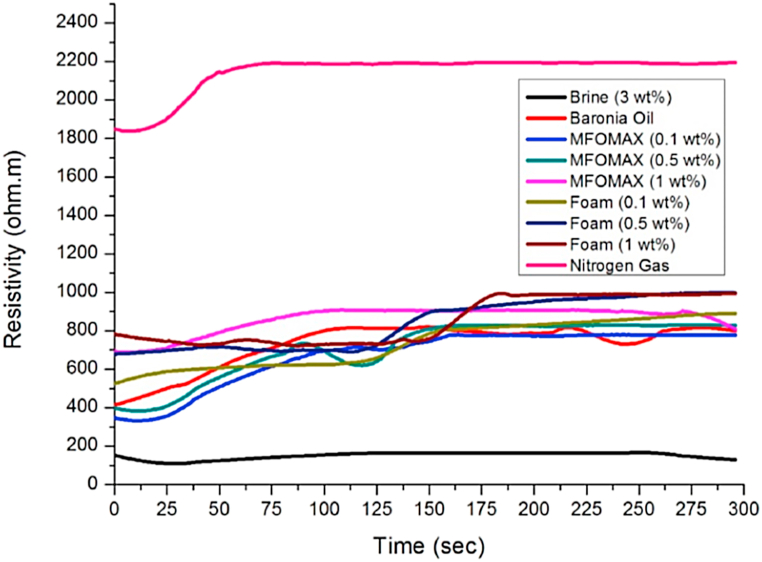
Fluid resistivity profile in dynamic condition.

As shown in Fig. 7, each fluid has a different range of resistivity values. The resistivity profile for each fluid in the static condition appears different from the values in dynamic conditions due to the results in the static condition only considering the internal molecules' fluid properties. In dynamic conditions, the resistivity results consider the interaction between the rock sample and the internal molecules' fluid properties. Therefore, the type of rock sample influences the resistivity results, as the rock sample contains electrical charges in the mineral composition. Results also show that nitrogen gas has the highest resistivity values, between 1800 and 2200 Ω m, and brine 3 wt% has the lowest values, between 100 and 200 Ω m. The MFOMAX and foam with various concentrations were plotted in the middle resistivity range of brine 3 wt% and nitrogen gas. The resistivity range profile for the MFOMAX alone with 0.1 wt% was within 320–732 Ω m, MFOMAX alone with 0.5 wt% was within 395–841 Ω m, and MFOMAX alone with 1 wt% was within 698–911 Ω m. The resistivity range obtained for foam using 0.1 wt% was within 532–957 Ω m, foam using 0.5 wt% within 699–1215 Ω m, and foam using 1 wt% within 703–1320 Ω m. These results were identified as the baseline for the resistivity profile for the foam propagation in the mixture of brine, oil, MFOMAX, and nitrogen gas at high temperature (90 °C).

### 5.2 Foam injection time towards resistivity measurement and water saturation

#### 5.2.1 Comparison of resistivity in foam propagation using three different surfactant concentrations

**Fig. 8:**
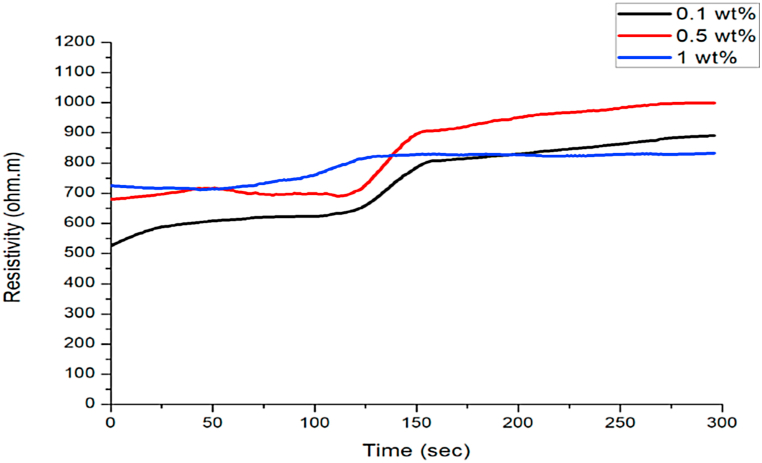
Comparison of resistivity towards time using MFOMAX 0.1 wt%, 0.5 wt% and 1 wt%.

Based on the results presented in Fig. 8, the impact of surfactant concentrations contributed to the resistivity measurement. This occurs as different surfactant concentrations have different surfactant properties, especially regarding the number of electrical charges contained in surfactant concentrations. The higher the surfactant concentrations, the higher the interactions between the electrical charges of the surfactant and the core sample. This causes the amount of free electrical charges captured in electrodes to be lower and reduces conductivity compared to the resistivity obtained in static conditions [58]. The use of a lower concentration led to lower adsorption. The results also indicate a limitation for adding MFOMAX as the foaming agent to generate foam in situ in the porous media.

#### 5.2.2 Quantification of three different surfactant concentration in water saturation

**Fig. 9:**
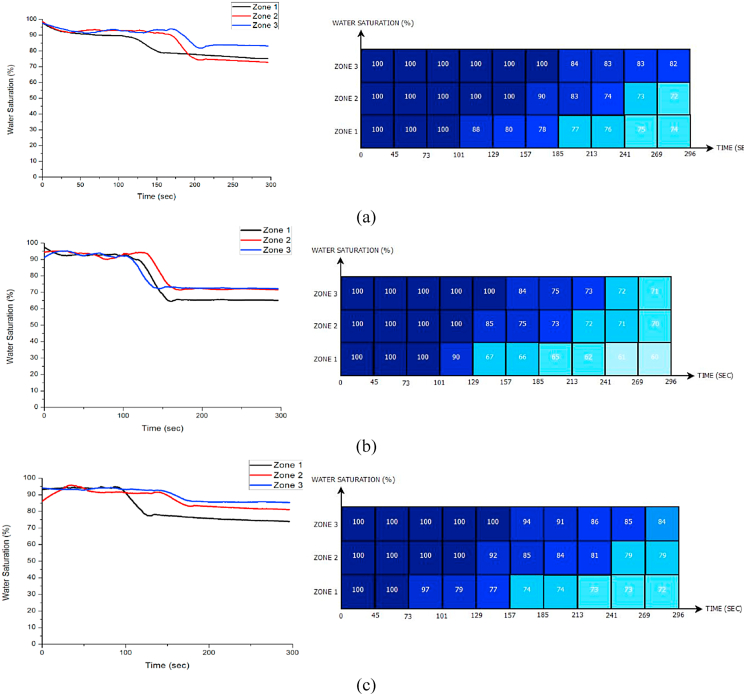
Water Saturation using MFOMAX surfactant (a) 0.1 wt% towards Time (b) 0.5 wt% towards Time (c) 1 wt% towards Time.

Fig. 9 (a) shows the quantification of decreasing water saturation and foam displacement as a function of time in three zones. The result shows that water saturation slightly decreased with time. The reduction of water saturation was 26 % in zone 1, 28 % in zone 2, and 18 % in zone 3. This indicates that the foam displacement using 0.1 wt% does not show prominent effects, and the formation water remains in the porous medium due to the early stage of foam disintegration. Fig. 9 (b) shows the water saturation reduction as a time function. Foam was able to sweep the formation fluid started from 73 s. Compared to the other zones, zone 1 shows the shortest propagation time. As foam has a higher concentration in this zone, this influences the behavior of the foam and its propagation. Higher surfactant concentrations thus affect foaming time. Based on the water saturation reduction, zones 1, 2, and 3 display a 28 %, 21 %, and 16 % water saturation reduction, respectively. Compared to the water saturation using 0.5 wt% MFOMAX, the reduction of water saturation, which leads to foam displacement efficiency, is considered the best concentration to sweep formation fluid in porous media.

The water saturation reduction using 0.5 wt% MFOMAX shows a good result, as shown in Fig. 9 (b). Regarding foam displacement efficiency, the foam consisting of 0.5 wt% reached the lowest water saturation compared to other surfactant concentrations (0.1 wt% and 1 wt%). The gap in water saturation reduction was 40 % in zone 1, 30 % in zone 2, and 29 % in zone 3. Zone 1 shows a greater reducing gap compared to other zones.

#### 5.2.3 Comparison of water saturation after water flooding and foam flooding with different surfactants

**Fig. 10:**
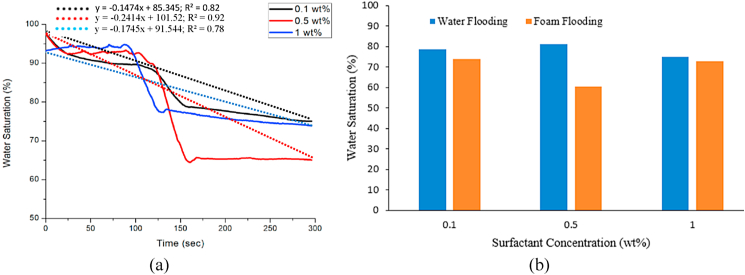
Comparison of water saturation at different surfactant concentrations and water flooding (a) water saturation comparison using MFOMAX 0.1 wt%, 0.5 wt% and 1 wt% (b) water saturation towards surfactant concentrations in comparison after water flooding and foam flooding.

Fig. 10 (a) shows that when using a surfactant concentration of 0.1 wt%, 0.5 wt%, and 1 wt%, the water saturation decreased to 74 %, 60 % and 72 %, respectively. As can be seen, the highest decrease in water saturation was not obtained for the highest surfactant concentration (1 wt%). The most efficient foam displacement occurred with 0.5 wt% of surfactant concentration. These results were related to the IFT between brine, oil, and surfactant. The higher the surfactant concentration, the lower the IFT value, indicating that the fluids become more miscible. When using MFOMAX as the surfactant, a maximum provision could be used. When the concentration of MFOMAX exceeded the maximum provision, it became more miscible. It created an emulsion between the oil and the surfactant, reducing the foam displacement. Using 1 wt% of surfactant concentration in the core flood caused a foam displacement reduction due to the emulsification between the oil and the surfactant. Based on the water saturation results, it can be concluded that foam is generated in zone 1. The water saturation reduction in zone 1 is decreased significantly compared to zones 2 and 3. Foam propagation occurs only in zone 1 for all three surfactant concentrations. Fig. 10 (b) compares water saturation reduction after water flooding and foam flooding in zone 1.

### 5.3 Foam propagation monitoring

Several parameters are important to observe when looking at foam propagation in porous media: foam propagation distance, foam velocity, and foam flow rate. This section compares the effects of three different surfactant concentrations on foam propagation distance, velocity, and flow rate.

#### 5.3.1 Resistivity towards distance

**Fig. 11:**
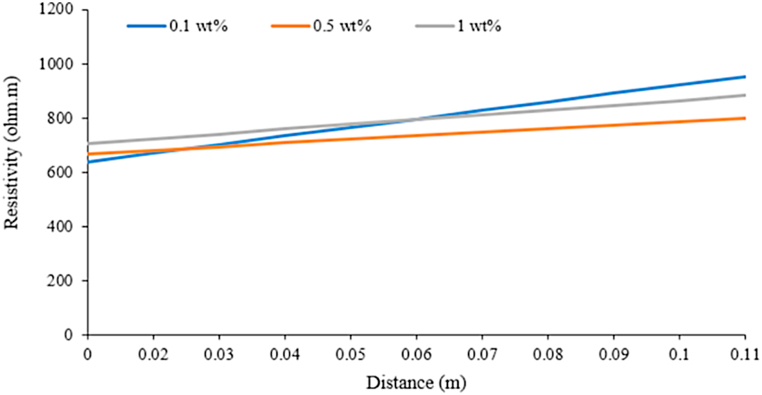
Resistivity towards distance using surfactant concentration 0.1 wt%, 0.5 wt%, 1 wt% in Zone 1.

Based on the resistivity and water saturation results, foam is seen to be generated and propagated in zone 1. The increasing surfactant concentrations affect foaming time and foam propagation time. These parameters lead to the porous media's foam velocity and flow rate. Still, none of the increasing surfactant concentrations significantly influenced the foam propagation distance, as shown in Fig. 11.

#### 5.3.2 Numerical analysis of resistivity measurement

**Fig. 12:**
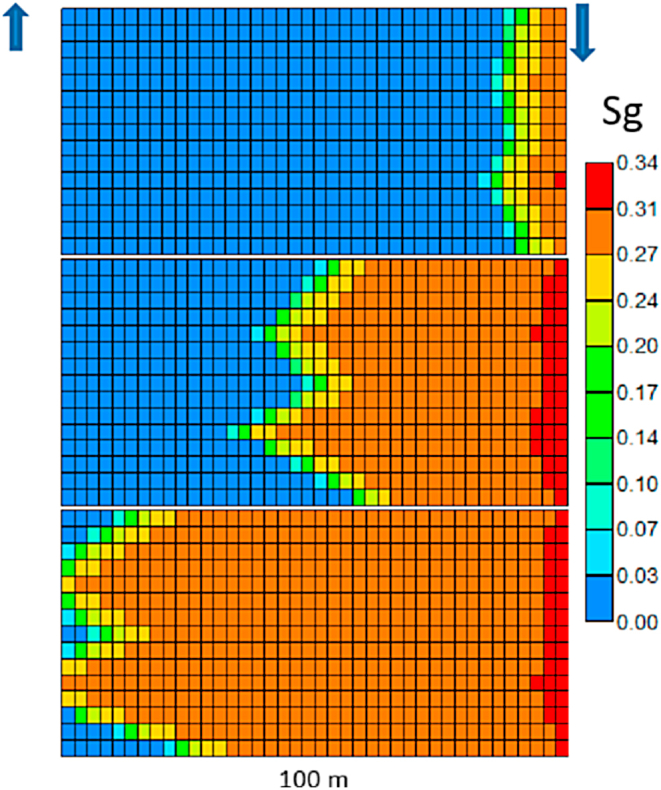
Gas saturation from foam injection.

A finite element numerical model of a Laterolog borehole probe used in practice is now described. Firstly, the electrode coefficient in a homogeneous medium is computed by using the finite element method (FEM). Secondly, an extensive numerical investigation is performed to determine the response of the foam front in a heterogeneous reservoir. Fig. 12 illustrates the foam flooding in a water-flooded reservoir. Pre-generated foam, consisting of N_2_ plus surfactant, is injected via the injector well. The average residual oil saturation after water flooding is 0.36. The foam collapses near the injector well and splits into gas and surfactant solution. As time progresses, the gas front propagates to the producing well. The permeability difference creates a non-uniform gas front. The water saturation from the foam simulation by STAR CMG is then exported to COMSOL to distribute the formation resistivity using Archie's equation. Fig. 12 shows the formation resistivity when the gas front reaches the producing well.

**Fig. 13:**
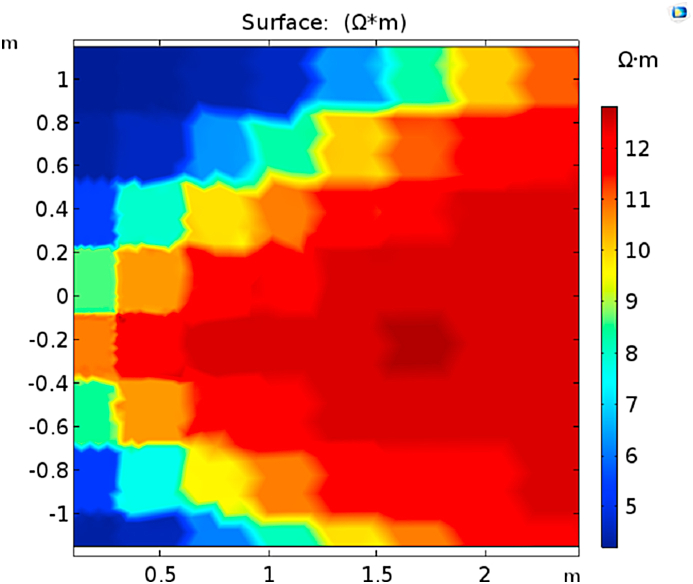
True formation resistivity.

Fig. 13 shows the current density distribution (colored), the potential field (contours), and the current lines emerging from the electrodes *Al*, *Am*, and *Au* in the homogeneous medium. The vertical invariability of the potential field surrounding the tool assures the focused current bunch of *Am* and the deep penetration. The apparent resistivity is calculated from the potential difference between the center electrode *m* and the reference electrode located at the surface. It shows the numerical test investigating the effect of reservoir heterogeneity when the gas front reaches the producing well. Electric currents will flow towards high water saturation, which is more conductive than gas fronts. Modeling shows water saturation measurement for the shale [59]. The existing analytical model can be tuned to apply to the sandstone reservoir with this data.

**Fig. 14:**
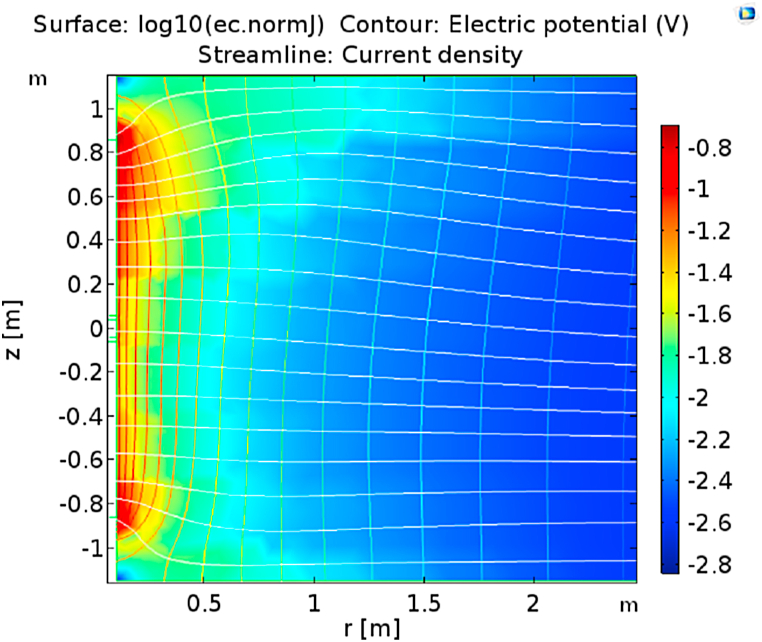
Normal current density and electric potential Rt from foam flooding.

The determination of the probe coefficient is shown in Fig. 14. In this case, the true resistivity is homogenous, Rt = 1 Ωm. The coefficient is the current and voltage ratio at the electrode's center, K = 0.146 m. The current density distribution and the potential field, as well as the current line emerging from *Al*, *Am* and *Au*. The apparent resistivity approximates the true resistivity, with small errors near the edges of the electrodes, which are insulators. The normal current density on the surface of the electrodes is not constant. When maintaining the equipotential of the electrodes, the current must intensify toward the edge.

#### 5.3.3 Foam velocity and foam flowrate

Foam velocity and foam flow rate are calculated to establish the foam behavior in the porous media. Foam flow rate is obtained by adjusting the gas and liquid flow rates accordingly. Based on the literature, the injection rate of the displacing fluid (foam) significantly affects the dynamics of the displacement in a porous media and the overall recovery of the displaced phase (oil) [48]. Surfactant concentration is the parameter that mostly affects the foam flow rate and velocity [49]. Table 8 shows the foaming time and foam propagation for increasing surfactant concentrations in the core flood experiment. Foaming time is the time taken for the foam generation in the porous media. As surfactant concentrations increase, the foaming time reduces because the volume and molecules in the foam booster are higher, which helps the generation of foam. Foam propagation and time differential are related to the foam stability in dynamic conditions (core flood conditions) due to the lamellae stability. Thus, when increasing the surfactant, the slowest foam propagation and time differential become higher due to the lamellae stability. Table 9 shows the influence of the increase in surfactant concentrations on the foam velocity and flow rate. Both foam velocity and flow rate gradually decreased as surfactant concentrations increased. These results indicate that for high surfactant concentrations, the number of surfactant molecules increases and causes the viscosity to increase. The gas has a very low viscosity compared with oil and water. However, when gas is in a dispersed phase, as in foam, its apparent viscosity is greatly increased, i.e., its mobility is greatly reduced. Hirasaki and Lawson [60] described a mathematical model for apparent viscosity in a smooth capillary tube for bubbles large enough that they travel sequentially along the tube. They found that foam texture is the most important variable affecting foam apparent viscosity in uniform, smooth capillaries. They also found that rock permeability and injected foam texture significantly affected foam propagation rate. In high permeability sandpacks (40–50 darcies), the foam propagated at the same rate as the liquid phase [61].

**Table 8:** Effect of increasing surfactant concentrations on foaming time and foam propagation.

Surfactant Concentration (wt %)	Foaming Time (sec)	Foam Propagation (sec)	Δt (sec)	Resistivity at 0 m (ohm.m)	Resistivity at 0.09 m (ohm.m)
0.1	120	147	27	639	891
0.5	118	153	35	667	774
1	101	165	64	706	847

**Table 9:** Foam velocity and foam flow rate as a function of surfactant concentrations.

Surfactant Concentration (wt %)	Foam Velocity (cm/s)	Foam Flow rate (cc/s)
0.1	0.061	0.69
0.5	0.059	0.67
1	0.055	0.63

The higher the viscosity of foam, the slower the foam propagates (velocity and flow rate). The lowest surfactant concentration creates the fraction of Nitrogen in the injection fluid to reduce and disperse the gas phase in the liquid phase. The liquid holds gas bubbles and reduces the sweep efficiency [11]. Foam increases the injected gas viscosity and the breakthrough time, improving the displacing efficiency. A simulation study with various considerations shows high low-foam and strong-foam propagation [62]. With the present in-depth experimental study, a generalized mechanistic model can be developed to determine the propagation form in the sandstone reservoir.

#### 5.3.4 Residual oil saturation

**Fig. 15:**
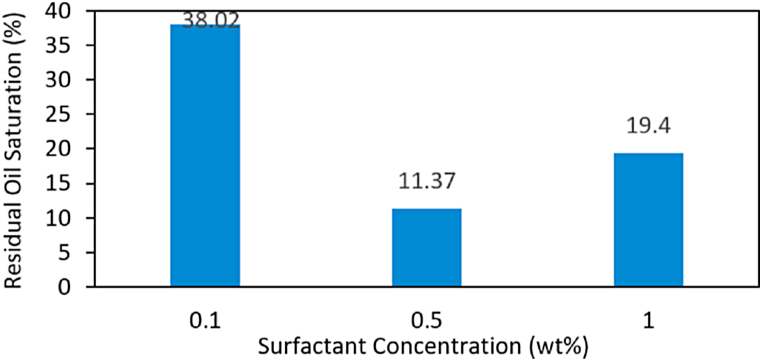
Residual oil after foam flooding.

Residual oil saturation is required to be a key factor in designing foam flooding. The results of residual oil saturation are depicted in Fig. 15, which shows a comparison of residual oil saturation at three different surfactant concentrations. The residual oil volume was calculated from the initial oil injected minus the oil volume produced (effluent) from forced displacement. Based on the results, the surfactant concentrations used in the experiment affected the residual oil saturation.

Among three surfactants, foam using 0.5 wt% surfactant concentration had the best results, indicating that 0.5 wt% had the lowest percentage of residual oil, approximately 11.37 wt%. The highest percentage of residual oil saturation was foam using 0.1 wt% surfactant concentration. The higher percentage of residual oil saturation in the rock surfaces wetted by oil will be less accessible to the surfactant to sweep oil to production. The lowest residual oil saturation obtained indicates that foam as a displaced fluid can sweep formation water, including oil, and affects the ease of foam propagation significantly. The lower the residual oil saturation obtained, the greater the oil produced. The increasing surfactant solution residual oil saturation decreases and enables the reduction of oil saturation in pore spaces [63]. The residual oil saturation decreased using 0.1 wt% to 0.5 wt% surfactant concentrations. In 1 wt% surfactant concentration, the residual oil saturation increased instead of decreased. The addition of surfactant in 1 wt% reached the maximum point, causing the emulsion to shift between displacing fluid and displaced fluid, which is indicated by the lowest IFT value obtained. The results show that foam using 0.5 wt% surfactant concentration was the optimum concentration to obtain the lowest reduction of residual oil saturation by reducing IFT between oil and water. Thus, it allows the foam to displace additional oil.

#### 5.3.5 Recovery factor

**Fig. 16:**
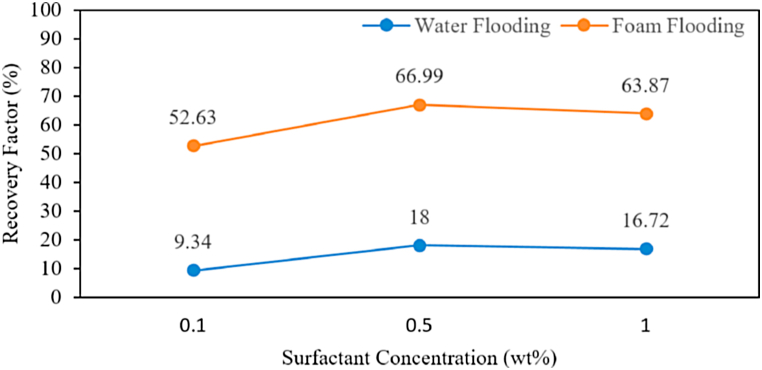
Comparison of Oil recovery for Water Injection and Foam Flooding.

The residual oil saturation is an influential factor in observing recovery factors during foam flooding. Fig. 16 shows the results of the comparison recovery factor after water flooding and foam flooding using three different concentrations. The results show that the recovery factor reached in foam flooding is higher than in water flooding. This is because water flooding has a large viscosity and density difference between displaced fluid and displacing fluid compared to foam flooding, which causes less recovery factor to be produced using water flooding. The addition of surfactant offers a higher recovery factor by reducing viscosity and density difference and reducing interfacial tension between displaced and displacing fluids. The improved recovery factor by reducing IFT is related to the residual oil re-mobilization after water flooding [64]. The recovery factor produced from foam flooding is dependent on the surfactant concentration. The recovery factor in foam flooding was 52.63 % at 0.1 wt%, 66.99 % at 0.5 wt%, and 63.87 % at 1 wt%. Past results shows that with increasing surfactant concentration, the recovery factor increased due to the reduction of IFT [65]. The IFT results observed during foam flooding were 0.5761 mN/m at 0.1 wt%, 0.3739 mN/m at 0.5 wt%, and 0.2027 mN/m at 1 wt%. The results show that the IFT decreased as surfactant concentrations increased. The recovery factor at 1 wt% decreased instead of increasing when surfactant concentration was increased. The reason is that the strong emulsion occurred at 1 wt% surfactant concentration, which made it difficult for foam to displace oil. Strong emulsification contributes to the oil recovery factor besides the effect of IFT reduction [66]. The strong emulsion reduces the oil recovery factor because the diameter of emulsions is large and blocks small pores; consequently, only partial residual oil was displaced, which reduced the oil recovery factor [67]. The strong emulsion is detrimental to improving the recovery factor in foam flooding.

Introduction of surfactant affect the production performance of the pore space and the addition of surfactant can be used for improving oil recovery. Overall, the results show that higher oil recovery obtained during foam flooding compared to water flooding and the selection of surfactant concentration in foam flooding is the important factor to be considered.

## 6 Conclusion

In the present study, foam was generated in-situ by alternating injection of nitrogen gas and surfactant solution of varying concentration (0.1 wt%, 0.5 wt%, and 1 wt%) followed by measurement of resistivity and water saturations in porous media during foam injection. The following are conclusions drawn from this study.

• With 0.1 wt%, 0.5 wt%, and 1 wt% surfactant concentrations, the resistivity ranges were 639–890 Ω m, 667–974 Ω m, and 706–846 Ω m, respectively. Thus, an increase in the surfactant concentration results in a higher resistivity. However, injected nitrogen gas has the highest resistivity values, between 1800 and 2200 Ω m, and the lowest resistivity range is 100–200 Ω m with no surfactant present.
• Resistivity is significantly increased when the foam reaches the first electrode and becomes stable after several minutes. The optimum surfactant concentration (0.5 wt%) generates up to 60 % reduction of the water saturation of its original saturation value in zone 1. The increasing surfactant concentrations did not affect the foam propagation distance. All three surfactant concentrations were ruptured in zone 2 and zone 3. Foam with the increasing surfactant existed in zone 1, which had 0.09 m.
• The increasing surfactant was followed by the decreasing foaming time, but the foam velocity and flow rate increased.
• Numerical methods were applied to calculate the probe coefficient of the three-electrode (laterolog tool) to determine the apparent resistivity obtained from the electric potential and the outflowing current of the measurement electrode.
• Involving three different surfactant concentrations in foam flooding, 0.5 wt% had the most prominent effect in terms of residual oil and oil recovery factor. Foam using 0.5 wt% reached 11.37 % residual oil saturation and 66.99 % oil recovery factor compared to other surfactant concentrations.

The main application of this research is for EOR applications to introduce foam flooding as one of the EOR methods, where resistivity is applicable to monitor and detect foam propagation in porous media. Simulation studies can be performed using present in-depth experimental results to determine foam propagation across sandstone reservoirs. Moreover, the presented resistivity measurement method can be applied to investigate water coning toward the production tubing and to select the proper layer for installation of water control Autonomous Inflow Control Device (AICD)/intelligent completion. Moreover, using this method, water saturation can be measured during hydraulic fracturing design to avoid water pollution by identifying the flow of fracking fluid (chemicals) to aquifers. Fracking should be performed by considering the water saturation distance to avoid polluting the surface water. For future studies, using a higher number of electrodes along the core sample is recommended to obtain a better precision of the resistivity measurement. In addition, the pressure gauge should be mounted in each electrode as the comparison method. Finally, the foam generation should be investigated by applying more than one cycle to quantify the effects of the number of cycles against the foam propagation in porous media.

## Additional information

No additional information is available for this paper.

## CRediT authorship contribution statement

**Javed Akbar Khan:** Writing – original draft, Validation, Software, Methodology, Investigation, Conceptualization. **Jong Kim:** Resources, Project administration, Funding acquisition. **Sonny Irawan:** Supervision, Resources, Project administration, Methodology, Funding acquisition. **Karina Aryanti Permatasar:** Methodology, Investigation, Formal analysis, Conceptualization. **Patrick G. Verdin:** Writing – review & editing, Methodology, Formal analysis. **Baoping Cai:** Writing – review & editing, Supervision, Formal analysis. **Nurudeen Yekeen:** Writing – review & editing, Visualization.

## Declaration of competing interest

The authors declare that they have no known competing financial interests or personal relationships that could have appeared to influence the work reported in this paper.
